# Back to the drawing board: Re-thinking the role of GLI1 in pancreatic carcinogenesis

**DOI:** 10.12688/f1000research.5324.2

**Published:** 2016-06-23

**Authors:** Tara L. Hogenson, Matthias Lauth, Marina Pasca diMagliano, Martin E. Fernandez-Zapico

**Affiliations:** 1 Schulze Center for Novel Therapeutics, Mayo Clinic, Rochester, MN 55905, USA; 2 Institute of Molecular Biology and Tumor Research, Philipps University, Marburg, 35043, Germany; 3 Department of Surgery, University of Michigan, Ann Arbor, MI 48109-5936, USA

**Keywords:** GLI1, Pancreatic Cancer, Carcinogenesis, Hedgehog, HH, pancreatic ductal adenocarcinoma, PDAC

## Abstract

Aberrant activation of the transcription factor GLI1, a central effector of the Hedgehog (HH) pathway, is associated with several malignancies, including pancreatic ductal adenocarcinoma (PDAC), one of most deadly human cancers. GLI1 has been described as an oncogene in PDAC, making it a promising target for drug therapy. Surprisingly, clinical trials targeting HH/GLI1 axis in advanced PDAC were unsuccessful, leaving investigators questioning the mechanism behind these failures. Recent evidence suggests the loss of GLI1 in the later stages of PDAC may actually accelerate disease. This indicates GLI1 may play a dual role in PDAC, acting as an oncogene in the early stages of disease and a tumor-suppressor in the late stages.

## Introduction

The protein GLI1, originally isolated in 1987 due to high levels of amplification in malignant glioma ( Kinzler *et al.*, 1987), is a member of the GLI family of transcription factors. This family also includes GLI2 and GLI3. The GLI family of transcription factors is highly conserved and is required for developmental response via transcriptional regulation of target genes ( Dennler *et al.*, 2007; Hui & Angers, 2011; Javelaud *et al.*, 2012). The GLI proteins, including GLI1, are transcriptional mediators of Hedgehog (HH) signaling, and regulate multiple cellular processes such as cell fate determination, tissue patterning, proliferation and transformation, which give this transcription factor a significant role in carcinogenesis if deregulated ( Dennler *et al.*, 2007; Hui & Angers, 2011; Javelaud *et al.*, 2012). GLI1 is expressed in different human malignancies including pancreatic ductal adenocarcinoma (PDAC) ( Eberl *et al.*, 2012; Fiaschi *et al.*, 2009; Goel *et al.*, 2013; Hui & Angers, 2011; Mills *et al.*, 2013; Rajurkar *et al.*, 2012; Thayer *et al.*, 2003). In PDAC, GLI1 is prevalently expressed in the stroma, in response to HH ligands secreted by the epithelial cells ( Yauch *et al.*, 2008). However, lower epithelial expression of Gli1 has also been reported, possibly with non-canonical functions ( Nolan-Stevaux *et al.*, 2009).

## GLI1 as an Oncogene in PDAC

GLI1 plays a key role in PDAC initiation by modulating the activity of two different cellular compartments, the epithelium and stroma. Rajurkar *et al.* demonstrated that targeted overexpression of GLI1 in the pancreas epithelium accelerates PDAC initiation by KRAS, a small GTPase mutated in more than 90% of PDAC cases ( Rajurkar *et al.*, 2012). Through use of a mouse model with simultaneous activation of oncogenic KRAS and inhibition of GLI1 in the pancreas epithelium, this group also demonstrated that decreased GLI1 activity reduced the incidence of KRAS-driven PDAC precursor lesions (pancreatic intraepithelial neoplasias or PanINs) and PDAC. Similarly, Mills and colleagues using a mouse model for pancreas-specific oncogenic KRAS expression (KC mice) bred on a *Gli1* null background (GKO/KC) defined a key role for GLI1 on PDAC initiation through the modulation of the activity of fibroblasts ( Mills *et al.*, 2013). The KC mice developed PanIN lesions with 100% penetrance and PDAC in advanced age, while the GKO/KC mice did not develop PDAC and had increased survival rate when compared to KC mice. Histopathological analysis of the pancreata showed KC mice developed PanIN lesions and PDAC, while 80% of GKO/KC had normal pancreata.

**Figure 1.**
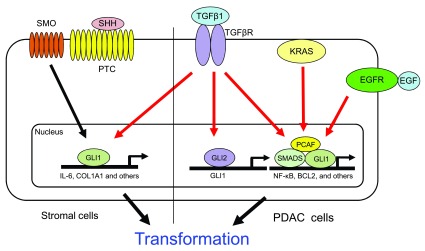
Schematic Representation of Pathways Modulating GLI1-expression/activity in Cancer. In canonical HH signaling in stromal cells, binding of the SHH ligand to Patched (PTC) activates the Smoothened (SMO) receptor, which induces GLI1 activation of its target genes, including IL-6 and COL1A1 (Martin E Fernandez-Zapico unpublished observation), leading to pre-malignant lesions. SMO-independent mechanisms for regulation of GLI1 in PDAC include KRAS, TGFβ, and EGFR. GLI1 has been shown to regulate the NF-κB pathway in a HH-independent manner downstream of KRAS, leading to pancreatic epithelial transformation. TGFβ promotes GLI2 expression in PDAC through Smad3 and β-catenin/LEF-TCF-dependent upregulation of GLI2 independent of HH signaling. TGFβ induced GLI2 expression, and subsequent GLI1 activation, is associated with EMT, tumor growth, and metastasis. TGFβ can also modulate GLI1 activity by promoting the formation of a transcriptional complex with the TGFβ-regulated transcription factors, SMAD2 and 4, and PCAF, at the BCL2 promoter in cancer cells to regulate TGFβ-induced gene expression. EGFR signaling is aberrantly activated in a majority of PDACs. EGFR and HH have been demonstrated to act synergistically to promote cancer cell initiation and growth by modulation of gene expression of distinct novel pathways through a GLI1-dependent mechanism.

**Figure 2.**
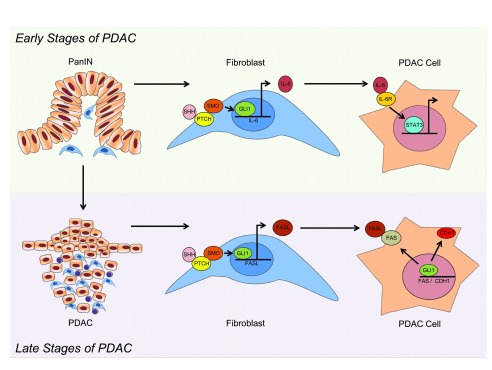
Working Model of the Dual Role of GLI1 at Different Stages of Pancreatic Carcinogenesis. During the early stages of PDAC, GLI1 is activated in the fibroblasts through canonical HH signaling. GLI1 promotes expression of the cytokine IL-6, which stimulates expression of STAT3 in neighboring cancer cells, promoting the progression of PanIN lesions to PDAC. In the later stages of PDAC, GLI1 binds the FASL promoter and regulates the expression of this ligand in the fibroblast, leading to lower levels of apoptosis in these tumors. In addition, in cancer cells, GLI1 induces the expression of FAS and CDH1 expression, leading to a tumor protective effect.

Analysis of the molecular mechanism underlying this phenomenon reveals that GLI1 both regulates different target genes and is modulated by different signaling pathways depending on the cellular compartment. For instance, GLI1 activity is mainly modulated by the canonical HH signaling in fibroblasts ( Figure 1) ( Yauch *et al.*, 2008). This cascade is activated by binding of the ligand to the receptor Patched (Ptch), resulting in activation of the G-coupled receptor, Smoothened (Smo) ( Javelaud *et al.*, 2012; Yauch *et al.*, 2008). Once activated, Smo induces GLI1 activation and upregulation of its target genes ( Hui & Angers, 2011; McMahon *et al.*, 2003). The HH ligand, Sonic Hedgehog (SHH), and components of the HH signaling pathway, including Ptch and Smo, are undetectable in the normal pancreas but overexpressed in PanINs and PDAC ( Thayer *et al.*, 2003). Inhibition of the HH pathway in PDAC cell-based xenograft models through Smo inhibition has been shown to reduce GLI1 activity and tumor growth ( Feldmann *et al.*, 2007; Thayer *et al.*, 2003; Yauch *et al.*, 2008). In addition, genomic sequencing of human pancreatic cancer samples revealed widespread mutations consistent with activation of the Hedgehog signaling pathway ( Jones *et al.*, 2008). While the association between HH activity and pancreatic cancer has been described over a decade ago, there is still uncertainty as to the downstream effect of HH activation in this disease. Mills *et al.* identified the cytokine IL-6 as a HH/GLI1 target gene in pancreatic fibroblasts ( Mills *et al.*, 2013). Increased IL-6 expression in the stromal compartment induces activation of STAT3 in the neighboring cancer cells, an essential molecular event for the progression of premalignant lesions in PDAC ( Figure 2).

## Hedgehog-Independent Mechanisms for GLI1 Expression in PDAC

While dysregulation of HH-GLI1 signaling has been shown to play an important role in PDAC formation, several studies have demonstrated that GLI1 expression can be activated through HH-independent mechanisms in PDAC, particularly in the epithelial compartment ( Dennler *et al.*, 2007; Eberl *et al.*, 2012; Goel *et al.*, 2013; Ji *et al.*, 2007; Nolan-Stevaux *et al.*, 2009; Nye *et al.*, 2014). Nolan-Stevaux *et al.* demonstrated that deletion of Smo receptor in pancreatic epithelium had no effect on KRAS induced tumor formation, nor on GLI1 expression in epithelial cells ( Nolan-Stevaux *et al.*, 2009). This indicates a Smo-independent mechanism for GLI1 regulation in PDAC cells downstream of KRAS. In fact, Ji *et al.* demonstrated that KRAS is a modulator of GLI1 activity and requires the transcription factor for PDAC growth *in vitro* ( Ji *et al.*, 2007). In the epithelial compartment, GLI1 is regulated in a HH-independent manner, downstream of KRAS. Accordingly, Ji *et al.*, showed that Gli1 protein degradation is blocked in a MAPK-dependent manner. Furthermore, Rajurkar *et al.* showed a role for GLI1 in the regulation of the NF-κB pathway, a signaling cascade linked to PDAC development ( Algül *et al.*, 2002; Ougolkov *et al.*, 2005; Pan *et al.*, 2008; Wang *et al.*, 1999), downstream of KRAS ( Figure 1) ( Rajurkar *et al.*, 2012). This group has identified the I-kappa-B kinase epsilon (IKBKE)/NF-κB pathway as a direct target of the GLI1 mediating KRAS-dependent pancreatic epithelial transformation *in vivo* (Junhao Mao, University of Massachusetts and Martin E. Fernandez-Zapico personal communication).

PDAC is characterized by a dense desmoplastic reaction associated with the primary tumor. The abundance of connective tissue is due to an increase in growth factor production in the tumor microenvironment through autocrine and paracrine oncogenic signaling pathways ( Mahadevan & Von Hoff, 2007). Oncogenic KRAS activates SHH production, but HH ligands do not activate the HH pathway in tumor epithelial cells in an autocrine manner ( Lauth *et al.*, 2010; Mills *et al.*, 2013; Yauch *et al.*, 2008). HH signaling in PDAC occurs in a paracrine fashion where HH signaling from PDAC cells to stromal cells has been shown to promote desmoplasia ( Yauch *et al.*, 2008). Lauth *et al.* demonstrated that this shift from autocrine to paracrine signaling is through activation of the RAS effector dual specificity tyrosine phosphorylated and regulated kinase 1B (DYRK1B) ( Lauth *et al.*, 2010). The authors proposed this is achieved through DYRK1B inhibition of GLI2 function and promotion of the repressor GLI3, and subsequent inhibition of GLI1, in PDAC cells.

TGFβ has been shown to promote GLI1 expression in pancreatic cancer cells ( Nolan-Stevaux *et al.*, 2009). TGFβ induces the expression of GLI1 through Smad3 and β-catenin/LEF-TCF-dependent upregulation of GLI2 independent of HH signaling ( Dennler *et al.*, 2007; Dennler *et al.*, 2009). Nye *et al.* demonstrated that TGFβ, in addition to controlling GLI1 expression, can also modulate its activity by promoting the formation of a transcriptional complex with the TGFβ-regulated transcription factors, SMAD2 and 4, and the histone acetyltransferase, PCAF, at the *BCL2* promoter in cancer cells to regulate TGFβ-induced gene expression ( Figure 1) ( Nye *et al.*, 2014). Activation of TGFβ induced GLI2 expression, and subsequent GLI1 activation, is associated with epithelial to mesenchymal transition (EMT), tumor growth, and metastasis ( Javelaud *et al.*, 2012). 

In addition to TGFβ and KRAS activation, epidermal growth factor receptor (EGFR) signaling, a cascade aberrantly activated in the majority of PDACs, has been demonstrated to play a critical role in HH/GLI1-regulated tumor-initiating pancreatic cancer cells ( Eberl *et al.*, 2012). Eberl and colleagues demonstrated EGFR and HH act together to promote cancer cell proliferation by modulating gene expression through a GLI1-dependent mechanism ( Figure 1). This suggests HH/GLI1 signaling works synergistically through distinct novel pathways during tumor initiation and growth.

## Clinical Trials Targeting the Hedgehog/GLI1 axis in PDAC

The concept that HH/GLI1 signaling might be required for PDAC growth, hence a suitable therapeutic target, has been first validated in a genetically engineered mouse model of pancreatic cancer that combines expression of oncogenic Kras with mutation of the tumor suppressor p53, the KPC mouse ( Hingorani *et al.*, 2005). Treatment of KPC mice with a Smo inhibitor in combination with gemcitabine led to a moderate but significant increase in survival ( Feldmann *et al.*, 2008; Olive *et al.*, 2009). A preclinical study of the HH inhibitor, saridegib (IPI-926), co-administered with gemcitabine, produced a transient increase in vascular density, increased chemotherapy drug delivery, and improved disease stabilization in pancreatic cancer cells ( Olive *et al.*, 2009). Based on these results, phase II clinical trials were approved evaluating saridegib and an additional Hh inhibitor, vismodegib (GDC-0449), for treatment of pancreatic cancer. Surprisingly, the clinical trial for both vismodegib and saridegib showed a higher rate of progressive disease when compared to placebo ( Catenacci *et al.*, 2013). Similar findings were seen in a separate phase I trial of vismodegib in 8 patients with pancreatic cancer ( LoRusso *et al.*, 2011). Although hedgehog inhibitors have been successful for treating basal cell carcinoma and medulloblastoma, they do not appear to have the same effect in advanced pancreatic cancer. 

These disappointing results left investigators questioning the molecular mechanism responsible for these failed clinical trials. Although there is overwhelming evidence that GLI1 plays an important role in tumor initiation and progression of several kinds of malignancies, these results suggest the transcription factor may have a tumor protective role in the later stages of certain cancers. In fact, recent studies investigating GLI1 expression in PDAC have revealed GLI1 may switch from a tumor promoting to a tumor protective molecule in the later stages of PDAC.

## GLI1 as a Tumor Suppressor in PDAC

In contrast to the current paradigm for GLI1 expression and tumor progression, one study found GLI1 expression may actually decrease cell motility in advanced PDAC ( Joost *et al.*, 2012). Joost *et al.* demonstrated that GLI1 regulates epithelial differentiation through transcriptional activation of the cell adhesion molecule, E-Cadherin (CDH1), in PDAC cells. Lowered expression of GLI1 in PDAC cells lead to a loss of CDH1 expression and promotion of EMT. The transition from epithelial to motile mesenchymal cells is thought to be a critical event for metastasis of carcinomas. Decreased expression of CDH1 is associated with increased metastasis and invasion, while increased expression is associated with lower tumor malignancy ( Seidel *et al.*, 2004; von Burstin *et al.*, 2009). PDAC is strongly associated with early invasion and metastasis. Loss of GLI1 was also shown to decrease expression of additional important epithelial marker genes, including *Keratin 19* ( *KRT19*) and adherens junctions components *EVA1* and *PTPRM*, leading to increased cell motility. This indicates that as PDAC progresses, lower GLI1 levels may actually prime tumor cells towards an EMT program, which would be associated with metastasis and advanced stages of the disease.

Mills *et al.* examined the role of GLI1 expression in the later stages of PDAC using a mouse model for advanced pancreatic cancer ( Mills *et al.*, 2014). In this study, the loss of GLI1 actually accelerated PDAC progression during the later stages of tumorigenesis. PDAC mice lacking GLI1 showed reduced survival when compared to GLI1 wild type littermates. While both cohorts of mice displayed the common features of advanced PDAC, loss of GLI1 was associated with decreased survival and increased tumor burden. Analysis of the mechanism revealed the pro-apoptotic FAS/FASL axis as a potential mediator for this phenomenon. Loss of GLI1 was associated with a significant decrease in expression of FAS/FASL, leading to lower apoptosis levels and increased tumor progression ( Figure 2).

In agreement with these findings, two recent studies demonstrated that the deletion of the GLI1 inducer SHH, using a mouse model for PDAC, led to more aggressive tumors ( Lee *et al.*, 2014; Rhim *et al.*, 2014). Interestingly, Rhim’s study reported the occurrence of poorly differentiated tumors, with increased vascularity, and significantly reduced stromal content. In contrast, the Lee paper only described a modest reduction in the stromal compartment. The current paradigm for PDAC is that the tumor stroma plays an important role in promotion of neoplastic growth and progression since PDAC is typically associated with a dense desmoplastic reaction. However, the Rhim study shows that tumors with reduced stroma may display a more aggressive behavior than those with an extensive stromal compartment. This concept is further supported by a recent report demonstrating that tumor stroma restrains pancreatic cancer progression and that pharmacological HH pathway activation in stromal cells can actually slow down *in vivo* tumorigenesis ( Lee *et al.*, 2014). The complexity of these findings reflects our incomplete understanding of the precise biological role of HH/GLI1 signaling in pancreatic cancer. In fact, the level of activation of HH signaling might induce different biological responses during the carcinogenesis process, as commonly observed during embryonic development. Manipulation of the membrane mediators of HH to reduce HH signaling leads to an increase of angiogenesis with low HH levels, but not with complete inhibition. Intriguingly, the Rhim and Lee studies generated a low HH signaling environment by eliminating SHH, but not IHH, another HH ligand expressed in pancreatic cancer. Similarly, studies altering the expression of Gli1 leave intact the other mediators of HH signaling, GLI2 and GLI3 (the latter mainly an inhibitor of HH target genes). It remains to be seen if manipulating GLI1 levels within the epithelial tumor compartment in later stages of disease is of any therapeutic value. Based on work from Fendrich *et al.* on HH signaling and acinar cell differentiation, it might even be provocatively proclaimed that increasing GLI1 levels could drive terminal differentiation and thus result in lower tumorigenicity ( Fendrich *et al.*, 2008).

## Discussion

These studies demonstrating GLI1 may act as a tumor suppressor in the late stage of PDAC give insight into the disappointing results of clinical trials testing HH inhibitors in metastatic PDAC patients. While the Olive experiments reported acute administration of IPI-926 increased survival due to decreased stromal content and increased vascularity, the HH inhibitor performed poorly in pancreatic cancer clinical trials in patients. One explanation for this discrepancy may be the short duration of treatment (3 weeks) in the Olive’s experiments, which may have not accurately detected disease progression following HH inhibition. This indicates that as PDAC progresses, the initial positive effects of HH inhibition may be eliminated as GLI1 levels decrease.

It is unclear why GLI1 levels decrease during PDAC progression *in vivo*. As previously discussed, one potential mechanism for lowered GLI1 expression in advanced PDAC may be due to activation of the kinase DYRK1B, which inhibits GLI1 expression through expression of the repressor GLI3 ( Lauth *et al.*, 2010). Activation of DYRK1B promotes a shift from autocrine to paracrine signaling in PDAC. This shift may lead to a decrease in GLI1 expression in PDAC cells. In addition, HH signaling promotes GLI1 expression in part through inhibition of GLI1 repressors ( Stecca & Ruiz, 2010). Decreased HH signaling in advanced PDAC may allow for increased expression of GLI1 repressors, such as GLI3 and Suppressor of Fused (Sufu), leading to decreased GLI1 expression and activity ( Stecca & Ruiz, 2010).

An alternative explanation for clinical trial failures of HH inhibitors in treatment of advanced PDAC may be due to GLI-independent effects of SMO inhibition, not necessarily due to a decrease in GLI1. Not all HH signaling responses are mediated by GLI transcription factors. SMO has been demonstrated to play a role in several cellular functions, including actin stress fiber formation, endothelial tubulogenesis, fibroblast migration, and regulation of glucose uptake independent of GLI transcription ( Brennan *et al.*, 2012; Chinchilla *et al.*, 2010; Teperino *et al.*, 2014). The failure of HH inhibition in PDAC could potentially be due to the loss of these GLI-independent SMO downstream effectors, while modulation of GLI1 expression may only be a secondary effect. In Rhim’s study, the authors discovered that SHH and GLI1 deficient tumors were more aggressive, poorly differentiated, and exhibited increased vascularity ( Rhim *et al.*, 2014). This suggests HH/GLI1 pathway inhibition may have a proangiogenic effect. Due to the increase in vascularity of the SHH deficient mice tumors, the authors investigated the effect of angiogenesis inhibition by administering anti-VEGF to tumor-bearing SHH deficient mice. This therapy led to a significant improvement in the overall survival of mice with undifferentiated tumors. Based on this response, the subset of PDAC patients with undifferentiated tumors may benefit from anti-angiogenic therapy.

In summary, due to the high complexity of PDAC initiation and progression, a personalized strategy for treatment should be considered. Under this strategy, PDAC should be analyzed before treatment to determine expression of GLI1 and upstream regulators in order to better define therapeutic options. Further studies are needed to fully understand the role of GLI1 in PDAC carcinogenesis. While there is evidence for a dual role of GLI1 in PDAC, this phenomenon has yet to be linked with other cancer types.
