# Supplementary material for: Back to the drawing board: Re-thinking the role of GLI1 in pancreatic carcinogenesis
Source: F1000Res. 2016 Jun 23;3:238. Originally published 2014 Oct 8. [Version 2] doi: 10.12688/f1000research.5324.2 (PMC4207242; doi:10.12688/f1000research.5324.2)
Supplement: Peer review history

# Peer review: Referee response for version 1

**DOI:** 10.5256/f1000research.5682.r6366

**Published:** 2014-10-23

**Authors:** Barbara Stecca

**Affiliations:** 1 Laboratory of Tumor Cell Biology, Core Research Laboratory-Istituto Toscano Tumori, Florence, Italy

This review provides a comprehensive summary about the role of the transcription factor GLI1 in pancreatic cancer (PDAC). This manuscript highlights the dual role of GLI1 during pancreatic carcinogenesis, acting as an oncogene in the early stages of disease and as a tumor-suppressor in the late stages. Recent evidence suggests the loss of GLI1 in the later stages of PDAC might accelerate disease progression. This might explain why Smoothened (SMO) inhibitors have been successful for treating basal cell carcinoma and medulloblastoma, but do not appear to have the same effect in metastatic PDAC. Moreover, this article summarizes recent data on the integration of GLI1 with other signaling pathways, suggesting that GLI1 is not only regulated by the upstream Hedgehog signaling in a SMO-dependent manner, but also by other oncogenic inputs, such as KRAS, TGF-beta and EGFR signaling.

Recent experimental data suggest that lower GLI1 levels associate with PDAC progression, whereas increasing GLI1 levels could drive terminal differentiation and decreased PDAC tumorigenicity. What is missing in this review is a consideration about the factors that might contribute to decrease GLI1 levels and activity during PDAC progression. I would also suggest to mention in the Discussion that the dual role of GLI1 is so far limited to PDAC, as the evidence is lacking in other cancer types.

I have read this submission. I believe that I have an appropriate level of expertise to confirm that it is of an acceptable scientific standard.

# Response

**Published:** 2016-06-14

**Authors:** Martin Fernandez-Zapico

**Affiliations:** Schulze Center for Novel Therapeutics, Mayo Clinic in Rochester, USA

1. *Recent experimental data suggest that lower GLI1 levels associate with PDAC progression, whereas increasing GLI1 levels could drive terminal differentiation and decreased PDAC tumorigenicity. What is missing in this review is a consideration about the factors that might contribute to decrease GLI1 levels and activity during PDAC progression.*
**Response: **We added additional considerations regarding other factors that may drive down GLI1 levels during PDAC progression to the “Discussion” section. Mechanisms included are activation of DYRK1B kinase, which promotes a shift from autocrine to paracrine signaling, which may lead to decreased GLI1 expression. Also, decreased HH signaling in advanced PDAC may allow for increased expression of GLI1 repressors.
2. *I would also suggest to mention in the Discussion that the dual role of GLI1 is so far limited to PDAC, as the evidence is lacking in other cancer types.*
**Response: **The following statement was added to the final paragraph of the Discussion section “Further studies are needed to fully understand the role of GLI1 in PDAC carcinogenesis. While there is evidence for a dual role of GLI1 in PDAC, this phenomenon has yet to be linked with other cancer types.”

# Peer review: Referee response for version 1

**DOI:** 10.5256/f1000research.5682.r6367

**Published:** 2014-10-21

**Authors:** Natalia Riobo

**Affiliations:** 1 Kimmel Cancer Center, Thomas Jefferson University, Philadelphia, PA, USA

The review is timely and addresses a very important problem in pancreatic cancer. The Smoothened inhibitors that work well for other tumor types have not only failed to stop the progression, but instead promote aggressive behavior in pancreatic cancer. The authors make a good case of balancing the evidence that suggests that there is HH-independent upregulation of GLI1 in the epithelial cells and a Hh-dependent upregulation in fibroblasts. Moreover, they nicely discuss how GLI1 is necessary for PanIN formation and then restrains further cancer progression. What is lacking in the review is a consideration of potential non-canonical effects of the Smo inhibitors. It is known that Smo induces cytoskeletal changes in fibroblasts, for instance, and that it can regulate glucose uptake in other cell types. Perhaps modulation of GLI1 is a bystander effect confusing the results. And the GLI1 knockout animals only partly acknowledge this interpretation, since some effects can be cell-type specific and opposing, as discussed in the review.

A minor criticism is the following: the introduction erroneously says that GLI1 is conserved from Drosophila to humans. However, GLI3 is the closest homolog in sequence and function to Drosophila Ci. It seems that the authors meant the GLI family is conserved.

I have read this submission. I believe that I have an appropriate level of expertise to confirm that it is of an acceptable scientific standard.

# Response

**Published:** 2016-06-14

**Authors:** Martin Fernandez-Zapico

**Affiliations:** Schulze Center for Novel Therapeutics, Mayo Clinic in Rochester, USA

1. *What is lacking in the review is a consideration of potential non-canonical effects of the Smo inhibitors. It is known that Smo induces cytoskeletal changes in fibroblasts, for instance, and that it can regulate glucose uptake in other cell types. Perhaps modulation of GLI1 is a bystander effect confusing the results. And the GLI1 knockout animals only partly acknowledge this interpretation, since some effects can be cell-type specific and opposing, as discussed in the review.*
**Response:  **Additional considerations were added concerning the use of SMO inhibitors and downstream GLI-independent effects to the Discussion section.
2. *A minor criticism is the following: the introduction erroneously says that GLI1 is conserved from Drosophila to humans. However, GLI3 is the closest homolog in sequence and function to Drosophila Ci. It seems that the authors meant the GLI family is conserved.*
**Response: **This statement was corrected to “the GLI family of transcription factors is highly conserved and is required for developmental response via transcriptional regulation of target genes” in the Introduction. The statement regarding Drosophila to humans was removed.

# Peer review: Referee response for version 1

**DOI:** 10.5256/f1000research.5682.r6370

**Published:** 2014-10-16

**Authors:** Alain Mauviel

**Affiliations:** 1 Team “TGF-β and Oncogenesis”, Centre de Recherche, Institut Curie, Orsay, France

In this article Hogenson *et al.* provide us with a timely review regarding the current knowledge about the role of the transcription factor GLI1 in pancreatic carcinoma. There is an important focus on the dual activity of GLI1 during pancreatic carcinogenesis depending on the stage of disease progression, as evidenced in most recent works in this field, both clinical and experimental, that are nicely summarized in this review.

Another important aspect of this review consists in integrating GLI1 as a transcription factor that is not solely regulated by Hedgehog signaling downstream of SMO but also by other pro-tumorigenic pathways, such as TGF-beta and KRAS signaling.

Overall, I believe that this manuscript is very focused and contains valuable information for the broader readership, with up-to-date citation of the most relevant and recent literature in the field.

A couple of minor points may be corrected or improved:

1. A figure summarizing the role of GLI1 downstream of the various pathways described to modulate its expression/activity would be helpful.
2. On page 3, one reads "TGFβ induces the expression of GLI1 through Smad3 and LET-dependent up regulation of GLI2". "LET-dependent" should be replaced by beta-catenin/LEF-TCF-dependent or something similar.

I have read this submission. I believe that I have an appropriate level of expertise to confirm that it is of an acceptable scientific standard.

# Response

**Published:** 2016-06-14

**Authors:** Martin Fernandez-Zapico

**Affiliations:** Schulze Center for Novel Therapeutics, Mayo Clinic in Rochester, USA

1. *A figure summarizing the role of GLI1 downstream of the various pathways described to modulate its expression/activity would be helpful.*
**Response: **An additional figure was added to the review (Figure 1) to describe the various pathways that modulate GLI1 expression as discussed in the review. Figure 1 from the first version of this article was changed to Figure 2.
2. *On page 3, one reads "TGFβ induces the expression of GLI1 through Smad3 and LET-dependent up regulation of GLI2". "LET-dependent" should be replaced by beta-catenin/LEF-TCF-dependent or something similar. *
**Response: ** “LET-dependent” was corrected to “β-catenin/LEF-TCF-dependent” on page 3.
